# Social desirability in personality inventories: Symptoms, diagnosis and prescribed cure

**DOI:** 10.1111/sjop.12015

**Published:** 2013-04

**Authors:** Martin Bäckström, Fredrik Björklund

**Affiliations:** Department of Psychology, Lund UniversityLund, Sweden

**Keywords:** Personality assessment, test items, self-ratings, social desirability

## Abstract

An analysis of social desirability in personality assessment is presented. Starting with the symptoms, Study 1 showed that mean ratings of graded personality items are moderately to strongly linearly related to social desirability (Self Deception, Impression formation, and the first Principal Component), suggesting that item popularity may be a useful heuristic tool for identifying items which elicit socially desirable responding. We diagnose the cause of socially desirable responding as an interaction between the evaluative content of the item and enhancement motivation in the rater. Study 2 introduced a possible cure; evaluative neutralization of items. To test the feasibility of the method lay psychometricians (undergraduates) reformulated existing personality test items according to written instructions. The new items were indeed lower in social desirability while essentially retaining the five factor structure and reliability of the inventory. We conclude that although neutralization is no miracle cure, it is simple and has beneficial effects.

## Introduction

Personality inventories have been criticized for being subjective to influence by response styles such as social desirability and acquiescence, thereby compromising the measurement of the trait-related contents of the included scales (i.e., the content validity). The present study concerns problems that occur when items are formulated such that raters have preference for the upper or lower part of the rating scale. It will be argued that item popularity, high as well as low, is a symptom of socially desirable responding. We propose a diagnosis of the problem and suggest a cure for coming to grips with it.

One problem with self-ratings of personality is the tendency of some respondents to react to the evaluative content of test items (Peabody, 1967). Arguably, the most basic diagnosis of items’ evaluativeness is how people in general tend to rate them. People generally agree on which items are desirable (e.g., Edwards, 1953; Konstabel, Aavik & Allik, 2006). We propose that this common perception within a population of what is desirable influences the mean rating level of some items, and also increases the risk of more or less deliberate socially desirable responding. Our basic model may be illustrated as follows: Two persons, Jack Enhancer and Jill Fair, have essentially the same level of Extraversion. When they rate themselves on a personality inventory Jack reads off not only the behavioral content of the items, but also how popular he feels that they are in the general population. Since he is an enhancer, he bases his ratings on both of these characteristics of the items. Jill, on the other hand, only reads off the behavioral content, and bases her ratings only on this. When Jack finds no hint of popularity in an item he rates at the same level as Jill. Put more technically, item popularity interacts with the enhancement factor and this makes the item ratings multifactorial. Our cure to this is quite simple: to rewrite items in a way that makes them more neutral and less obviously popular in a population.

The study concerns a specific quality of personality items, not raters. Accordingly, the results will not bear on a population of humans, but rather a population of items (personality items). To validate the importance of item popularity we will relate it to another quality, namely social desirability. If within a sample of items (from a population of all possible items) there is a correlation between the mean rating level and the extent to which the item is related to social desirability (a separate quality), then mean rating level may be problematic. It may make ratings multifactorial, namely, partly driven by social desirability, partly by personality content.

Since the present study concerns graded response scales the term “rate” will be used instead of “endorse,” which is more suitable for dichotomous no/yes scales. Based on similar research on dichotomous scales (Edwards, 1953; Wahler, 1965), we hypothesize that the mean level of item ratings in a questionnaire is critical for whether it will have a factor related to social desirability. Items should be more strongly related to desirability if their responses deviate from the midpoint of the response scale. Items with high mean ratings (after reversing negative items) are by definition more popular in the population. This quality of the item, irrespective of the specific content, should tend to drive responses in the same direction because some subjects prefer to rate themselves more desirably. If this is so, all measures having high mean ratings will be related to one another, not because of an inherent feature of the different traits, but since the items that we use elicit the motivation to respond in a socially desirable manner. If such items are distributed over all scales of an inventory then all scales will be correlated (the orthogonality problem, see for example, Saucier, 2002). This does of course not exclude that there may be other reasons why measures of personality factors correlate with one another, for example, they may in fact not be completely independent (Block, 1995). But since this study concerns items, not persons, we leave this possibility out for now.

What is the current evidence that item popularity constitutes a problem in standard personality inventories? A look at the norm data from the NEO-PI-R (Costa & McCrae, 1992) and the HEXACO-100 (Lee & Ashton, 2004) reveals that the mean ratings of many of their scales and subscales deviate substantially (almost 1 SD) from the midpoint of the rating scale. This indicates that the items of these inventories generally are subject to item popularity.

We argue that a graded (Likert format) response scale is subject to popularity-related item rating effects, just as a dichotomous (true/false) response scale is, but have found no previous studies of this (Konstabel *et al*., 2006, for a clearly related but different approach). Although it is conceivable that previous results could be directly generalized to Likert scales, empirical study is necessary to determine to which extent this is so, and to what extent the relation to social desirability is best described as linear and/or quadratic. Modern personality inventories differ from older ones not only in response format, but also in that the item selection more often is based on an explicit theoretical model rather than simply empirical (based on a Principal Component Analyses – PCA).

It should be noted that it is not self-evident that high mean values are the ones that have the strongest relation to social desirability. But the evaluative quality of an item, its popularity, can influence its rating, increasing the probability of a high mean rating. Some items have negative connotations, others are neutral, and yet others have positive connotations. Popular items tend to have a clearly evaluative content, that is, they tend to be concerned with the question of whether one is “good” or “bad.” What we propose is that the evaluative quality of an item interacts with social desirability, such that participants who tend to describe themselves in a positive way will find items with a positive evaluative quality more attractive. The gut reaction to this statement may be that it is tautological, but since item rating in this study is about mean levels, and social desirability (in this context) is about variation, it is not. Item rating levels in Likert scales will be related to social desirability concerns and this tendency in some raters can produce a factor that is positively related to all scales of the Five Factor Model (FFM).

## Study 1

The first study aims at showing the positive relationship between item mean rating and item social desirability. Edwards (1953) found a very high correlation for this relation and Kuncel and Tellegen (2009) showed that item social desirability is not linearly related to the mean rating. They suggested that very high and very low rating levels are associated with lower rated social desirability. In the present study we will analyze the items from another perspective.

Test constructors often aim for a balance between positive and negative items in their scales. This means that, for example, high item ratings sometimes reflect extraversion and sometimes introversion. The present study does not concern effects of item balancing. All negative items will be reversed, as it is the mean rating level given that the direction of measurement is the same for all items of a scale that is of interest (e.g., for all items, high ratings reflect Extraversion). We propose that the tendency to score high on social desirability measures, suggesting that the respondent is an enhancer, interacts with the popularity of the test item, that is, participants who score high on social desirability are especially prone to give high ratings to items that are generally scored high by other subjects.

We also investigate the relation between the loadings to the first principal component (PC) and mean rating level. If there is substantial common content in many items (many items with obvious popularity), irrespective of the factor that they measure, then it is highly likely that this common content will show up as high loadings to the first PC from a PCA based on all items (e.g., Bäckström, 2007; Edwards & Edwards, 1991).

### Method

#### Participants and procedure

The samples consisted of three sets of Swedish speaking participants. The first had 1,698 participants, 32.8% males and 67.2% females (about 24% did not report their sex), with a mean age of 29 years (*SD* = 9.0). The second had 1,388 participants, 31% males and 69% females (8% did not report their sex), with a mean age of 30.7 years (*SD* = 10.6). The third had 878 participants, 32% males and 68% females (about 11% did not report their sex), with a mean age of 30.2 years (*SD* = 10.0). Most participants were spontaneous visitors to an Internet site (http://www.pimahb.com) and all volunteered without compensation. After the participants were finished rating, their results were presented to them together with brief information on the scales.

#### Materials

Two FFM-inventories from the International Personality Item Pool (IPIP) archive were used. The first one (Goldberg, 1999; Goldberg, Johnson, Eber *et al*., 2006) was created to mimic the original NEO PI R developed by Costa and McCrae (1992). The second (dataset 2) was the 486-item IPIP-AB5C inventory (Hofstee, de Raad, Goldberg, 1989; Goldberg, 2006) validated in Bäckström, Larsson, and Maddux (2009). The third (dataset 3) was Goldberg’s personality markers (Goldberg, 1992), with 100 adjectives divided evenly over the FFM scales. The inventories were translated into Swedish and back-translated by a professional translator. All scales have a very high reliability (α = 0.80–0.92).

Furthermore, two scales on social desirability from the “Personal Attribute Survey” of the IPIP archive were included. They measure Impression Management (18 items, α = 0.87) and Self-deception (10 items, α = 0.87), and have been found to correlate highly with Paulhus’ (1984) original Balanced Inventory of Desirable responding (BIDR, Paulhus, 1984) (*r* = 0.84–0.86; Bäckström, unpublished data). The Self-deception and Impression Management scales do not provide pure measures of social desirability, but are suitable indicators of it for the present purposes.

### Preliminary analysis

What, then, is item popularity when the rating scale has a Likert format? Most Likert scales use a format with 3 to 9 scale steps. On a five point (0–4) Likert scale, as was used in the present study, an item can be defined as popular if it is has a mean rating above the middle step (2). For a rating above the midpoint to appear, it should be the case that high scores on the scale are related to social desirability, as it is conceived in society. An unpopular item, accordingly, has a mean rating below the midpoint of the rating scale. However, if the item is reversely coded, then item unpopularity will also be reversed.

Mean ratings (after reversing all items scored in the opposite direction) were calculated, to be used as the items’ popularity index. This was the dependent variable of this study (note that N is the number of items, not the number of persons). Each inventory was subjected to Principal Component Analyses (PCA), extracting the first component only. The loadings to the first PCs were used as an indicator of the amount of general item commonality, irrespective of personality traits, namely, the general commonality index of the items.

Then the items’ social desirability values were calculated by correlating the items with the two measures of social desirability (Self Deception and Impression Management). The correlations were used as indicators of the strength of the relation to standard social desirability measures, which were the social desirability indices of the items.

Our hypothesis states that an item’s social desirability index and its general commonality index are related to the popularity index of the item, namely, that items with high mean ratings (popular items) are generally more strongly related to typical indices of an individual’s level of social desirability and the item’s general commonality.

### Results

The first inventory to be analyzed was the IPIP-NEO (using data set 1). The mean rating level after reversal of negative items was 2.56, which is 0.56 points above the scale midpoint (2), suggesting that the items were generally rated somewhat off the midpoint of the response scale. The *SD* of the items’ mean ratings was 0.50 and it is this variability that is hypothesized to correlate with the items’ social desirability indices. Correlations were 0.37 (*p* < 0.001), 0.31 (*p* < 0.001), and 0.50 (*p* < 0.001) for Self Deception, Impression Management, and the first PCA component, respectively. These correlations suggest a moderate relation between the mean rating level and the item social desirability indices. In other words, if an item had a high mean rating participants with high scores on the social desirability measures tended to rate it relatively higher; a *de facto* interplay between mean item rating and participant level of social desirability. In other words, our suggested diagnosis of what causes socially desirable responding was supported by the data.

The correlations between the items’ mean rating and the social desirability indices were estimated separately for each FFM-scale (left panel of Table 1). The highest correlations were found for Extraversion and Agreeableness. Since there was some variation in the strength of correlation across scales we also estimated the partial correlation, controlling for the scale that the item belonged to. First the items’ scale belongingness was dummy coded into four variables. These four variables were then used in calculating the partial correlations, which were 0.54 (*p* < 0.001), 0.41 (*p* < 0.001), and 0.30 (*p* < 0.001) for Self Deception, Impression Management, and PCA1, respectively. In other words, the results show that when analyzed within each factor, the item’s popularity is even stronger correlated with social desirability.

**Table 1:** Correlations between the items’ mean ratings and their social desirability indices, for each FFM scale across three inventories

Inventory	IPIP-NEO PI			AB5C			Marker scales		
			
Scale	SfD	IM	PCA1	SfD	IM	PCA1	SfD	IM	PCA1
Extraversion	0.62	0.46	0.74	0.59	0.78	0.84	0.47	0.68	0.72
Agreeableness	0.75	0.59	0.82	0.28	0.39	0.58	0.31	0.66	0.61
Conscientiousness	0.20	0.23	0.47	0.39	0.42	0.59	0.24	0.41	0.53
Emotional stability	0.52	0.19	0.46	0.32	0.42	0.56	0.80	0.83	0.93
Openness	0.26	0.34	0.46	0.44	0.39	0.53	0.60	0.62	0.78

*Note:* All correlations except 0.24 for Marker scales are significant at the *p* = 0.001 level; SfD = Self Deception; IM = Impression Management; PCA1 = First principal component

The next inventory to be analyzed was the AB5C (using dataset 2). The correlation between mean item ratings and the items’ social desirability indices in this inventory were 0.22 (*p* < 0.001), 0.28 (*p* < 0.001), and 0.42 (*p* < 0.001) for Self Deception, Impression Management and PCA1, respectively. The partial correlations controlling for the scale that each item belonged to were 0.50 (*p* < 0.001), 0.39 (*p* < 0.001), and 0.62 (*p* < 0.001), for Self Deception, Impression Management and PCA1, respectively. The mean item ratings of the Marker scales revealed moderate to high correlations to the items’ social desirability indices; they were 0.42 (*p* < 0.001), 0.51 (*p* < 0.001), and 0.62 (*p* < 0.001), respectively. The partial correlations controlling for scale were 0.59 (*p* < 0.001), 0.65 (*p* < 0.001), and 0.78 (*p* < 0.001), respectively.

The above results support the hypothesis that the mean item rating level is linearly related to social desirability. This does not exclude a quadratic relation, which was tested and found to be significant in all inventories (see left part of Table 2). The strongest quadratic relations were found for Self Deception and PC1, whereas for Impression Management they were non-significant in most cases. The quadratic relation sometimes added as much as 5.9% to the explained variance of the linear relation. This suggests that desirability is somewhat reduced at the very high mean rating level. As it is possible that the non-linear relation was affected by a restriction in range in the tails of the distribution, the right part of Table 2 displays the same linear and quadratic relations when item rating variability (*SD*) was controlled for. As could be expected the relations were somewhat weaker, but several of the quadratic relations remained significant.

**Table 2:** Linear and Quadratic relations between mean level of item ratings and the social desirability indices

Inventory	SfD-Mean	IM-Mean	PCA1-Mean	SfD-Mean *SD* Corr.	IM- Mean *SD* Corr.	PCA1- Mean *SD* Corr.
IPIPNEO-Linear	0.27** 14.3%	0.27* 10.0%	0.42** 25%	0.194* 11.7%	0.140* 6.3%	0.130* 3.6%
IPIPNEO-Quadratic	–0.26** 5.7%	–0.11 0.9%	–0.20** 3.1%	–0.26** 3.8%	–0.186* 2.2%	–0.095 0.6%
IPIP-AB5C-Linear	0.11** 4.8%	0.24* 7.8%	0.32** 18.3%	0.185** 8.5%	0.206* 6.6%	0.322** 20.4%
IPIP-AB5C-Quadratic	–0.26** 5.9%	–0.09 0.6%	–0.26** 5.9%	–0.176** 1.9%	–0.084 0.4%	–0.213** 2.9%
Markers-Linear	0.33* 17.4%	0.57 30.1%	0.49** 38.0%	0.511** 22.0%	0.634 24.9%	0.647** 40.5%
Markers-Quadratic	–0.17 2.1%	0.03 0.0%	–0.24* 4.2%	–0.055 0.1%	0.178 1.3%	–0.014 0.0%

*Notes*: * = *p* < 0.05, ** = *p* < 0.001. Upper values are standardized B coefficients in the model with both linear and quadratic terms using standardized independent variables. Percentages indicate explained variance, for the Quadratic term unique contributions are displayed. SD Corr. – Linear and quadratic predictors are corrected for the item endorsement standard deviation; SfD = Self Deception; IM = Impression Management; PCA1 = First principal component

### Discussion

The results from Study 1 show that the rating level of the item is a relevant factor also in modern personality inventories (with graded response scales). This was found in three different kinds of Five factor inventories, and in all cases it was rather strongly (linearly) related to our measures of social desirability. In addition to the linear relationship there was also a quadratic relationship which suggests that items with very high rating levels were rated somewhat lower by subjects who scored high on social desirability scales (see also Kuncel & Tellegen, 2009). The study thus firmly established that there is both a linear and quadratic trend between item mean ratings and social desirability. The relation was stronger for some factor scales; for example the Extraversion scale items had stronger correlations than what was found for all items in the inventories taken together (see Johnson, 2004, for a similar result). However, the findings suggest that overall, items that are highly popular (after reversal of negative items) introduce a social desirability factor into the scales. The clear relationship between item popularity and social desirability strengthens our claims that test constructors who are concerned with socially desirable responding should turn their attention to popular test items. Popular items are easy to identify, and the fact that item popularity is symptomatic for items that tend to elicit socially desirable responses is good news for those of us who aim for purer measures of personality content. Furthermore, our diagnosis of the causes of socially desirable responding, that is, the interaction between popularity level and enhancement motivation, was also supported by the results. This diagnosis is the inspiration to the suggested cure, to which we turn next.

## Study 2

What, then, would be a possible cure of the “desirability disease,” that is, the contamination of social desirability in self-reports? Peabody (1967) suggested that responses to personality items may have both a content-related component and a desirability-related component. Using sophisticated methods for test construction, with scales based on a valence-balanced set of items to handle problems with acquiescence social desirability, Peabody (1967) could show that it is possible to separate content from evaluation. Our suggested cure is inspired by Peabody’s, but is simpler. We suggest that part of the treatment may consist in constructing evaluative neutralized items, that is, reducing the popularity of existing items by rephrasing them in a way that brings their mean rating level closer to the midpoint of the scale.

This is basically the method that was proposed in the study by Bäckström, Björklund and Larsson (2009). But Study 2 expands on this by examining the feasibility of the neutralization method, and clarifying its relation to item popularity. We propose that it should be possible, without too much effort, to generate a pool of rephrased items, to formulate a set of selection criteria, and use these criteria to select those items that are less socially desirable than the original but still can be expected to load on their respective FFM-factor. One way of testing whether a method is simple is to try it on novices. If psychometrically unsophisticated people such as, say, undergraduate psychology students, are able to neutralize personality items, the method should qualify as simple. Remember that it is important that the neutralization of items only affects the evaluativeness/popularity of the item, so that it still captures the trait that it is supposed to. Otherwise the reliability and validity of the inventory, such as the factor structure, will be impaired. But if the social desirability of the undergraduates’ new items is lower while at the same time retaining the desired properties of the inventory, we will have shown that our neutralization method can be formulated in a way that even laymen are able to understand and follow. In our view, this would be equivalent to an easily administrated cure.

Study 2 was divided into three phases. In the first phase students rephrased items from the IPIP-100. In the second phase the new items were rated with respect to their social desirability. Finally, in the third phase an Internet sample rated themselves on the new items as well as the original IPIP-100 items. Our hypothesis was that the new inventory, based solely on items neutralized by laymen, would capture the same five factors as the original inventory (be strongly correlated at the factor level), and also be less affected by item popularity than the original inventory (be less correlated with measures of social desirability, and have a relatively smaller first PC).

### Method

#### Participants

Eighty-eight (18 men and 67 women, 3 failed to report sex) undergraduate students of psychology were recruited to construct new items. Mean age was 25.1 with a range from 19–46. Some of them did not succeed in constructing enough items, resulting in a group of 70 subjects for the item construction phase.

The newly constructed items were rated for social desirability. There were 72 participants (53 women, 19 men), all undergraduate clinical psychology students. Mean age was 24.7 years with a range from 20–42.

Finally, 127 participants (44 men, 83 women) rated themselves on the items of the new and the original inventory, to enable later evaluation of test validity. The sample consisted mainly of undergraduate students who were asked to visit the Internet site (http://www.pimahb.com), but a few of them were spontaneous visitors. Mean age was 28.4 with a range from 17–65.

### Materials

#### Item neutralization

The original items were taken from the IPIP-100 (Goldberg *et al*., 2006), an FFM inventory consisting of 100 items, 20 from each factor. To make the task less taxing for the participants they each modified only ten items, and from only one of the factors. In other words, different participants modified items from different factors. Altogether, 700 new items were constructed.

#### Item desirability rating

Six inventories were created including between 118–120 items each.

The following criteria were formulated for the item selection, 607 unique items being found to fulfill the criteria:

1. It must be a statement, not a question
2. It should refer to personality, not to other kinds of traits, for example, attractiveness or intelligence.
3. The items should not be reversed (e.g., from an extraversion original to introversion)
4. It should be comprehensible and grammatically correct

The selected 607 unique items were randomly mixed with the 100 original items. Participants were asked to rate the desirability of the items on a five point Likert scale.

#### Personality self-rating

A new inventory was created from items that were found to have significantly lower rated social desirability (compared to the original item), in all 288 out of the 607. The inventory consisted of 99 randomly selected items, 20 from each of the five factors (for one factor there were only 19 viable items). In addition, the 50 items from the original inventory were included, resulting in a total of 149 items for the participants to rate (in a random order).

#### Procedure

The instructions for the item neutralization stated that social desirability is a problem in personality inventories and that subjects rating personality test items tend to react to the desirability of the item. They then asked participants to rephrase the 10 items by making them more neutral (less desirable, or more if the item was reversed), and provided some four tangible tips on neutralization:

1. Construct an item that you would find less desirable yourself.
2. If the adjective is evaluatively positive, use a less evaluative one, or rephrase in a way that makes the adjective less evaluative.
3. Do not change an item from positive to negative (direction).
4. Think of whether the item is reversed or not.

The last part of the instruction included one example where the item “Love to help others” was changed to “Have a need to help others.” The participants were allowed 15 minutes to construct the 10 new personality items. In the *Item desirability rating* as well as the *Personality self-rating* phase the participants read a short standard instruction and then simply went on to make the ratings.

### Results

Our first hypothesis was that our lay psychometricians would be able to construct less socially desirable items than those from the original inventory. The pair-wise difference between the 99 included items and each counter-part item from the original inventory showed that 93 of them had a significantly (*p*s < 0.05) lower mean score, providing firm support for the hypothesis.

The 10 items from each factor that received ratings closest to the midpoint of the rating scale were selected to be included in the new inventory. The five new scales were subjected to standard item-analysis, for example, reliability analysis with Cronbach alpha and item-total correlation, and since some of the items had very low item-total correlation, two items from each scale were excluded, resulting in, five final scales, with eight item each. The reliabilities of these scales were α = 0.73, 0.52, 0.81, 0.70, and 0.85, for Extraversion, Openness, Conscientiousness, Agreeableness and Emotional stability, respectively (as compared to 0.86, 0.70, 0.84, 0.88 and 0.88 for the original inventory).

The next step was an exploratory factor analysis. To increase the reliability of the observed variables in this analysis the 40 items were randomly aggregated into 20 parcels. The same aggregation was conducted for the original inventory (using 40 randomly selected items). Each parcel consisted of two items. The number of extracted factors was forced to be exactly five. Using these specifications, the total variance explained was 61.4% and 71.0%, for the new and the original inventory, respectively. However, the first PC explained only 19.8% of the variance in the new inventory, while it explained 27.8% in the original, constituting firm support for the hypothesis that neutralized items result in a smaller first PC (suggesting less common variance). For all FFM-factors except Agreeableness, the items loaded uniquely on the correct factor.

Further support was revealed by a mean correlation between the scales of 0.09 in the new inventory, and 0.27 in the original. The correlations (Table 3) between the scales measuring the same factor were, 0.81, 0.64, 0.75, 0.84, and 0.60, for Extraversion, Openness, Conscientiousness, Agreeableness, and Emotional stability, respectively (corrected correlations in parentheses). Taking attenuation due of unreliability into account, only Emotional stability had a somewhat weak correlation.

**Table 3:** Correlations between scales of the original inventory (upper right), of the new inventory (lower left), and between inventories (diagonal)

	E	O	C	Es	A
Extraversion	0.81	0.59	0.32	0.27	0.49
Openness	0.32	0.64	0.02	0.10	0.15
Conscientiousness	0.13	–0.27	0.75	0.28	0.25
Emotional stability	0.21	–0.04	0.09	0.84	0.20
Agreeableness	0.25	–0.22	0.16	0.08	0.60

*Notes*: N = 127. Correlations higher than 0.18 are significant at *p* < 0.05.

To summarize the results, our lay item constructors were able to construct useable items resulting in FFM scales with impressive correlations to the scales of the original inventory, but with a smaller first PC and a lower intercorrelation between scales.

Our last hypothesis concerned a weaker correlation for the relation between the scales of the neutralized version (as compared to the original) and the measures of social desirability. Table 4 displays the correlations and reveals that the hypothesis was supported regarding some of the scales. There were lower correlations to four out of five scales, especially to self-deception. Note that the neutral inventory actually had a stronger correlation between emotional stability and self-deception, indicating less support for the hypothesis for this scale.

**Table 4:** Correlations between FFM scales and measures of social desirability for the new and the original inventory

Factor	New		Original	
	
	SfD	IM	SfD	IM
E	0.35 (0.42)*	0.19 (0.24)	0.51 (0.59)	0.22 (0.08)
O	0.22 (0.33)**	–0.17 (–0.26)	0.38 (0.49)	0.01 (0.02)
C	0.05 (0.06)**	0.18 (0.22)**	0.29 (0.34)	0.41 (0.43)
Es	0.66 (0.77)	0.35 (0.43)	0.58 (0.67)	0.35 (0.41)
A	–0.00 (0.00)**	0.36 (0.47)	0.29 (0.34)	0.49 (0.58)
Mean	0.24	0.18	0.43	0.28

*Notes*: Figures in parentheses are correlations corrected for attenuation. SfD = Self Deception; IM = Impression Management. N = 127, correlations higher than 0.18 are significant at *p* < 0.05; ** = significantly lower correlation in the new inventory (one-tailed, *p* < 0.05); * = trend for lower correlation in the new inventory (one-tailed, *p* < 0.10).

### Discussion

Study 2 set out to test the hypothesis that neutralization is a simple potential cure to the problem with social desirability in personality items. The strategy was to look out for item popularity, which had been shown in Study 1 to interact with social desirability. Ratings of the items’ social desirability supported the notion that popular items are in fact more socially desirable. More importantly, it was found that it is relatively easy to construct items that are less desirable. And if it is easy, why should we keep items that are popular (scored relatively high on the rating scale) in our personality inventories? Instead, item popularity may be used by test constructors as a heuristic for identifying problematic items, which may be deleted, replaced with better existing items, or why not modified by means of the evaluative neutralization method.

The most important part of the study was also the most risky, in terms of being tough to our proposed method. Is it really possible for lay people to construct 10 usable test items (in 10–15 minutes), and that these, provided some item selection based on standard scale construction techniques, could be put together to a personality inventory on par with inventories constructed by professionals, and even better when it comes to scale independence? Although we are inclined to answer in the affirmative, it should be duly noted that the patient could not be declared fit after just one treatment. We are the first to admit that evaluative neutralization is no miracle treatment, but would like to interpret the findings from Study 2 as evidence of its positive effects. For example, the new inventory had scales with rather high reliabilities, and the exploratory factor analysis clearly supported four out of five factors of the FFM. PCA showed that the new inventory’s first PC was much smaller, supporting our hypothesis that evaluatively neutralized items have less common variance. This was further corroborated by less correlation between scales in the new inventory. The fact that there was about 8% difference between the first PC of the new and the original inventory, and about 9% difference in total explained variance, rules out lower reliability in the new items as a possible explanation of the effects. This indicates that the four following PCs of both inventories included about the same amount of systematic variance. The very high correlation between the new and original scales further supports the new inventories construct validity. Finally, the generally lower correlation between the scales and typical measures of social desirability is encouraging.

On the downside, there were still correlations between scales from different factors in the new inventory. Whether such correlation is due to flaws related to the method of evaluative neutralization or due to shared content between factors of the FFM cannot be established by the present research. A more interesting problem is the correlation between emotional stability and social desirability. It has been suggested that social desirability measures tap into both bias and content (originating from the FFM factors), and that it therefore cannot be concluded from such measures whether emotional stability still has a large component of social desirability or if self-deception measures emotional stability. Correlation between emotional stability and the evaluativeness of personality items has been shown before (Borkenau & Ostendorf, 1989), so one possible interpretation is that measures of emotional stability, at least partly, tap the same kind of processes as are behind item popularity, social desirability, and evaluative aspects of personality.

## General Discussion

Many attempts of investigating item social desirability have been based on ratings by laymen (e.g., Johnson, 2004). The present study shows that item social desirability is indicated by the mean rating level alone. This is useful news (and at the same time a reminder from the old days, e.g., Edwards, 1953; Wahler, 1965) for those who want to reduce the influence of social desirability in their inventories (which is not a given, c.f. McCrae & Costa, 1983). The results supported the hypothesis that the mean level of an item’s rating is moderately to strongly related to how much the item correlates with social desirability.

What are the specific implications of the current findings for the construction of personality items and inventories? It seems safe to assume that test constructors prefer respondents to base their ratings on the extent to which the test items correspond to their perceived level of factors such as Extroversion or Openness, rather than on how popular the items are in the population. Popular items will trigger social desirability concerns and result in scales where content and evaluation are mixed. Therefore neutral items, where the population tends to provide mean ratings that are close to the midpoint of the typical Likert scale will, according the present results, involve less evaluation. Checking for popular items may prove a helpful heuristic tool in scale construction.

What is so important with pure measures of personality, one may ask? Our response is that our proposed method for making personality measures purer may not be of immanent need for those with applied interests, such as recruiting job candidates. It is possible that tests with evaluatively loaded items are superior to our neutralized version when it comes to selection, for example. But to the extent that it would be possible to predict work performance with independent measures of personality content and evaluation the situation may change. Once validated versions of pure measures exist, they should be evaluated in applied settings. In the meantime, those of us who are interested in personality *per se*, namely, do basic research on personality structure and the like, will continue to strive for purer measures. This is so since they allow better testing of theoretical models of personality. Firmer conclusions can be drawn when the fit between data and model can be accounted for by variance in the relevant constructs, rather than measures that are confounded by socially desirable responding.

However, one of the chief advantages of including items that receive high or low mean ratings is that they make it possible to capture high and low levels of a trait. According to Item Response Theory, scales should be composed of items that reliably measure specific levels of the trait (e.g., Stark, Chernyshenko, Drasgow & Williams, 2006). An item to which almost nobody agrees strongly would, if rated highly, indicate a very high level of the trait (and vice versa for not agreeing). Variability at that level would increase the possibility of discriminating between people at the high level of the scale. But if content and evaluation is mixed in these items, due to their popularity, this road to better scale construction seems closed. Therefore, we suggest that instead of constructing items that differ in mean rating level, items that measure the same trait but differ in context or behavior should be constructed. The reason for using the graded Likert scale is to enable the rater to indicate a high or a low level of the personality trait (its intensity). If a more fine grained discrimination of levels is desirable, then a more fine grained Likert scale should be used.

Test development requires probing new techniques, and unwillingness to abandon preexisting instruments or kinds of measures may prove costly for personality psychology. The IPIP is a laudable initiative in this regard; items have been developed to help researchers develop new instruments. Ironically, however, it would be unfortunate if items for new inventories were sampled exclusively from the IPIP pool, since this would restrict the possibility of handling social desirability. This is a challenge for future test constructors.

One avenue for future scale construction is to construct items that vary in evaluative content. This is exactly what was suggested by Peabody over forty years ago (Peabody, 1967). He proposed that by combining adjectives that vary in the evaluative aspect, but are constant in the descriptive aspect, it should be possible to develop scales that are free from the evaluative aspect. The problem with this method is that it has been difficult to find both positive and negative terms to describe the same personality traits. The three steps (neutralization, desirability rating, self-rating) used in Study 2 are considerably simpler, and still have beneficial effects.

From a more general point of view, the present results are related to all kinds of research and evaluations that are based on self-ratings. For example, in surveys where attitudes are inquired into, item-popularity related social desirability should be at least as common as when asking about personality related behavior. As for explicit measures of stereotypes and prejudice, for example, racism scales, it has been suggested (Fazio & Olson, 2003), that people control their responses in the direction of what is politically correct (social desirable). To remedy this, a whole research field has evolved where attempts are made to measure attitudes by more indirect methods, for example, the well-known Implicit Association Test (Greenwald, McGhee & Schwartz, 1998). In later years such techniques have been used also to measure personality, and especially the FFM (Boldero, Rawlings & Haslam, 2007; Grumm & von Collani, 2007). This kind of work is very promising and gives hope of finding methods to complement self-ratings. On the other hand, self-ratings as a method of finding out what people do, feel or think, is very likely to be one of the main methods also in the future. Improvements such as evaluative neutralization are valuable due to the extensive use of self ratings, not least in important areas such as selection and recruitment. Therefore, further development of the self rating method is crucial for the field of personality, and probably for other fields of psychology too.
